# Transport properties of mechanochemically synthesized copper (I) selenide for potential applications in energy conversion and storage

**DOI:** 10.1186/s11671-024-04025-5

**Published:** 2024-04-30

**Authors:** Marcela Achimovičová, Katarína Gáborová, Jiří Navrátil, Petr Levinský, Olha Skurikhina, Juraj Kurimský, Jaroslav Briančin, Tomáš Plecháček, Dáša Drenčaková

**Affiliations:** 1 Institute of Geotechnics, Slovak Academy of Sciences, Košice, Slovakia; 2 Institute of Metallurgy, Technical University of Košice, Košice, Slovakia; 3 Faculty of Chemical Technology, University of Pardubice, Pardubice, Czech Republic; 4 Institute of Physics, Czech Academy of Sciences, Prague 6, Czech Republic; 5 IREC, Barcelona, Spain; 6 Faculty of Electrical Engineering and Informatics, Technical University of Košice, Košice, Slovakia

**Keywords:** Copper selenide, Milling, Conductivity, Thermoelectrics, Sodium-ion battery

## Abstract

This work studied the thermal stability, electrical, and thermoelectrical properties of copper(I) selenide, Cu_2_Se synthesized by high-energy milling in a planetary ball mill. The phase composition was investigated by X-ray powder diffraction analysis and scanning electron microscopy. The conversion of the precursors during mechanochemical synthesis and the stability of the product was monitored by thermal analysis. The dependence of electrical properties on the product porosity was observed. For the densification of Cu_2_Se, the method of spark plasma sintering was applied to prepare suitable samples for thermoelectric characterization. High-temperature thermoelectric properties of synthetic Cu_2_Se were compared to its natural analogue-mineral berzelianite in terms of its potential application in energy conversion. Based on the results a relatively high figure-of-merit, ZT parameter (~ 1.15, T = 770 K) was obtained for undoped Cu_2_Se, prepared by rapid mechanochemical reaction (5 min). Cyclic voltammetry measurements of Na/NaClO_4_/Cu_2_Se cell implied that mechanochemically synthesized Cu_2_Se could be used as a promising intercalation electrode for sodium-ion batteries.

## Introduction

Copper (I) selenide Cu_2_Se is an interesting p-type semiconductor for its numerous potential applications such as solar cells, thermoelectric converters, photodetectors, superionic materials, optical filters, photovoltaics, and ion batteries [1–5] due to its thermal stability, electrical and thermoelectric properties. Cu_2_Se exists even at room temperature in different crystallographic modifications, including orthorhombic, monoclinic, and cubic structures, depending on the preparation method. Lévy-Clément and co-workers have prepared cubic Cu_2_Se by chemical bath deposition on an inert Pt-substrate from a selenosulfite-containing bath at 75 °C. By electrochemical transformation, its orthorhombic phase at room temperature was achieved [6]. A monoclinic structure was reported by Murray and Heyding [7], while Stevels and Jellinek documented an orthorhombic structure of Cu_2_Se [8]. These copper (I) selenide investigations and references belong to the period of 70–90 years of the last century. Later in 2011–12, Gulay and co-authors reported the monoclinic phase at room temperature [9], and Liu and co-workers cubic anti-fluorite Cu_2_Se structure at 450 K [10]. The Cu_2_Se preparation methods as a solvothermal [11], hydrothermal [12, 13], microwave-assisted hydrothermal method [14, 15], electrodeposition [16], chemical bath deposition [17], chemical synthesis reduction [18, 19], thermal evaporation [20], magnetron sputtering [21], solid-state reaction with pulsed laser deposition [22], microwave heating [23], and classical melting, annealing or sintering [24–26] were used in the last 10 years. However, most of these methods of Cu_2_Se preparation needed expensive equipment or a complicated post-treatment process. For the first time in 2012, Yu and co-authors prepared tetragonal α-Cu_2_Se by subjecting the Cu and Se starting materials to high-energy milling in a Spex mixer mill for between about 10–30 h, and milling speed > 1000 rpm [27, 28]. Later in 2015, Gahtori et al. synthesized nanostructured Cu_2_Se during 50 h of milling with a speed of 400 rpm [29], and Butt et al. during 5 h of milling with a speed of 450 rpm in planetary ball mills [30]. They used the milling technique to generate diverse nanoparticles from starting materials for subsequent consolidating of the product nanoparticles under pressure and temperature to examine thermoelectric performance. Recently, mechanochemical synthesis for the preparation of nanostructured orthorhombic modification of Cu_2_Se after 5 min of milling in a planetary ball mill was realized and appealed extensive attention due to the simple, one-pot, solvent-free and very fast synthesis [31]. The electrical properties, thermoelectric performance, and thermal stability of such mechanochemically synthesized copper(I) selenide have not yet been investigated.

In this paper, just the abovementioned properties of mechanochemically synthesized Cu_2_Se were studied in order to obtain new and unique knowledge about the potential use of this advantageously very fast-prepared semiconductor material in the energy conversion and storage field. The high-temperature thermoelectric performance of natural Cu_2_Se- mineral berzelianite was also measured and compared to its synthetic analogue. The properties of synthetic Cu_2_Se were studied by several characterization techniques, evaluated, and compared with reported copper selenides prepared by more laborious and costly methods.

## Materials and methods

Mechanochemically synthesized Cu_2_Se was prepared by milling in a laboratory planetary ball mill Pulverisette 6 (Fritsch, Germany) according to the conditions: loading of the mill-50 balls (10 mm in diameter), the material of the milling chamber and balls-tungsten carbide, the volume of the milling chamber-250 ml, the mass of Cu powder-3.08 g, the mass of Se powder-1.92 g, ball-to-powder ratio 73:1, milling atmosphere-Ar, rotation speed-550 rpm, and milling time-5 min [31].

Natural Cu_2_Se (Chelopech, Bulgaria) contained cubic Cu_2_Se (berzelianite; ICDD-PDF2 01-072-7490) and traces of CaCO_3_ (calcite; ICDD-PDF2 01-071-3699) see Fig. 4. For thermoelectric measurements, 5 g of the sample was treated by dry milling for 5 min in the same mill type and conditions as mentioned above.

The X-ray diffraction analysis (XRD) was carried out using a D8 Advance diffractometer (Bruker, Germany) with Bragg–Brentano geometry and Cu_Kα_ as a radiation source. The Diffracplus Eva tool and the ICDD-PDF 2 database were utilized for phase identification.

Thermoanalyzer STA 449 F3 Jupiter (Netzsch, Germany) was used for differential thermal analysis (DTA) under dynamic conditions in argon (50 cm^3^ min^−1^) and a heating rate of 10 °C min^−1^.

Scanning electron microscopy (SEM) observations were made with a MIRA3 FE-SEM microscope (TESCAN, Czech Republic) with an energy-dispersive X-ray (EDX) detector (Oxford Instrument, UK).

A standard four-point probe method with a test head (Ossila Ltd., UK), and an electrical source were used to study the electrical properties of Cu_2_Se [32, 33]. The preparation of Cu_2_Se tablets weighing 0.37 g was carried out by pressing with a laboratory hydraulic tablet press (Specac, USA), at a pressure of 1 and 2 t, without retention time, and at room temperature. The round pressed tablets had a diameter of 7.06 ± 0.01 mm with a density reported in the literature of 6.9–7.0 g.cm^3^. To obtain reproducible results, the probe tips were fixed in a constant position (the distance between them was 1.27 mm) and loaded with a constant contact force [34].

The thermoelectric properties of natural Cu_2_Se and its synthetic analogue were studied on 10 mm round pellets with a thickness of 4 mm sintered by the spark plasma sintering (SPS) in graphite matrices at 500 °C, under the pressure of 50 MPa with holding time 10 min. After SPS, the pellets were polished and cut into geometrically suitable pieces for further measurements. The electrical resistivity, *ρ* and the Seebeck coefficient, *S* were measured by the four-terminal static direct-current method, using an LSR-3 m (Linseis, Germany) at temperatures from 300 to750 K. For the determination of thermal diffusivity, α in the temperature range from 100 to 700 K the laser flash apparatus LFA 427 (Netzsch, Germany) was used. The specific heat capacity, *c*_*p*_ was measured by a comparative method using Inconel-718 alloy as a reference. Subsequently, thermal conductivity, κ was calculated according to the formula $\kappa =\alpha .{c}_{p}.\varrho$, where ρ is the density of the sample.

Cyclic voltammetry (CV) and galvanostatic cycling with potential limitation (GCLP) experiments were carried out with an MPG-200 potentiostat/galvanostat (BioLogic Science Instruments SAS, France) at room temperature. In an Ar-filled glove box, the Swagelok cells were prepared from a mixture of Cu_2_Se: polyvinylidene fluoride: carbon in an 8:1:1 ratio cast on etched Al-foil as a working electrode and a Li-sheet as a counter and reference electrodes. As an electrolyte, 1 mol NaClO_4_ was used in a non-aqueous solution of ethylene carbonate and dimethyl carbonate in a volume ratio of 1:1. The cells were cycled between 1.5 and 2.5 V vs. Na^+^/Na and for GCLP charge–discharge measurements between 1.5 and 2.4 V vs. Na^+^/Na at a current density of 10 mA.

## Results and discussion

### Completeness of mechanochemical synthesis and thermal stability of the Cu_2_Se

**Fig. 1:**
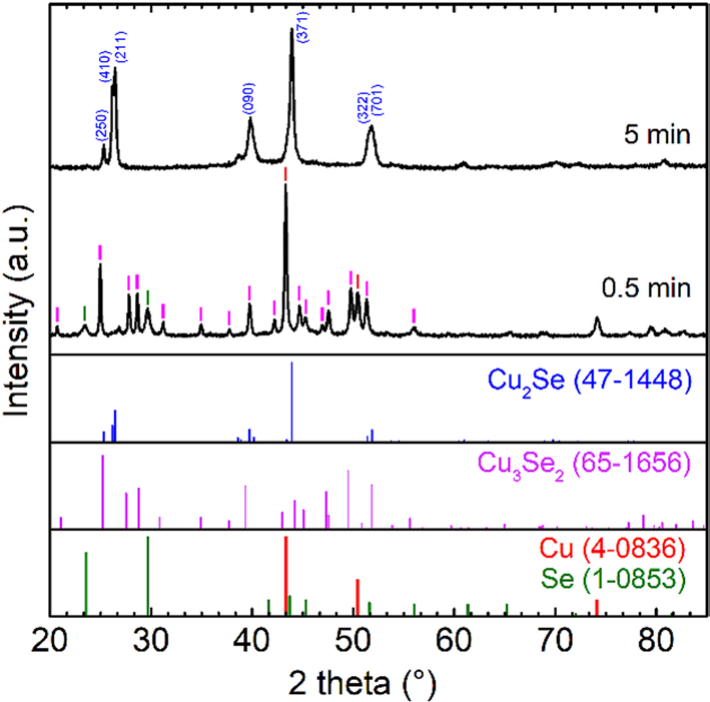
XRD patterns of 2Cu/Se mixtures milled for 0.5 and 5 min; reference patterns of Cu and Se precursors, Cu_3_Se_2_ and Cu_2_Se

Copper (I) selenide was prepared by mechanochemical synthesis with a milling time of 5 min, which was confirmed by the XRD pattern in Fig. 1. Unreacted elemental precursors Cu and Se were not present in the product Cu_2_Se with orthorhombic structure (ICDD PDF 47-1448), as shown by their reference patterns. The sample milled for 0.5 min contained, in addition to unreacted precursors, a Cu_3_Se_2_ phase (ICDD PDF 65-1656) as an intermediate product of the mechanochemical synthesis of Cu_2_Se which gradually disappeared with increasing milling time. The kinetics of this mechanochemical reaction, together with the proposed mechanism and refined structure of the Cu_2_Se product, has recently been described in detail and published by [31].

**Fig. 2:**
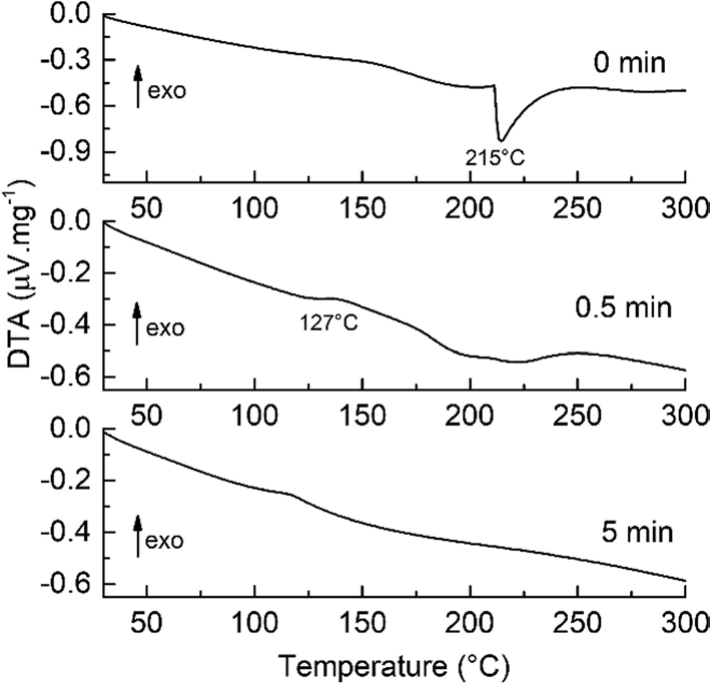
DTA curves of unmilled and milled 2Cu/Se mixtures for 0.5 and 5 min

The unmilled and milled 2Cu/Se mixtures for 0.5 and 5 min were subjected to thermal analysis to monitor the conversion of precursors during mechanochemical synthesis DTA curves of 2Cu/Se mixtures with the various times of milling are shown in Fig. 2.

Only one thermal effect was visible in the DTA curve of the unmilled mixture of the precursors. The endothermic peak at 215 °C corresponds to the melting of hexagonal selenium as described in our earlier research of mechanochemically synthesized bismuth selenides [35]. The reduction of this DTA signal was more pronounced after 0.5 min of milling. The traces of the melting of unreacted selenium can be observed. A slight sign of an exotherm at ~ 127 °C is probably related to the transformation of a very small amount of low-temperature (LT) Cu_2_Se synthesized by milling alongside the predominant intermediate Cu_3_Se_2_ phase (see Fig. 1). According to Gulay and co-authors, the LT modification of Cu_2_Se is transformed to the high-temperature (HT) modification above ~ 400 K [9]. After 5 min of milling, no peaks and thus no thermal transformations were detected in the DTA curve, which means that HT modification of Cu_2_Se was prepared by mechanochemical synthesis with complete conversion of precursors to the final product and agreed with the XRD pattern in Fig. 1. The negligible indirectness of the DTA curve was due to the residual moisture in the product. In addition, it was clear from thermal measurements that mechanochemically synthesized Cu_2_Se was stable up to 300 °C.

### The study of the electrical properties of synthesized Cu_2_Se

As the representative engineering parameter, the porosity of potential electrode materials is important. The porosity of Cu_2_Se was varied by changing the pressure of the tablet press from 1 to 2 t (see Table 1). When the pressure of 2 t was applied the porosity decreased by 9%. Therefore, the electrical properties of mechanochemically synthesized Cu_2_Se product depending on the porosity were also experimentally investigated. Namely, sheet resistance, resistivity, and conductivity were derived from the measurement data population consisting of 200 values for each Cu_2_Se product with different porosity. The resulting values are in Table 1.

**Table 1:** Electrical properties of Cu_2_Se products with different porosity and thickness depending on the pressure of the tablet press

Product/pressure	Thickness (mm)	Porosity (%)	Sheet resistance (Ω/square)		Resistivity (mΩ m)		Conductivity (S/m)	
			Mean	Standard Dev	Mean	Standard Dev	Mean	Standard Dev
Cu_2_Se/1 t	1.69 ± 0.02	21	16.42	2.21	27.75	37.28	36,641.43	4545.49
Cu_2_Se/2 t	1.52 ± 0.02	12	4.47	1.58	6.80	2.40	161,653.73	44,835.96

The conductivity of Cu_2_Se increased 4.4-fold due to a decrease in the porosity of the material by applying a pressure of 2 t in preparation of the tablets. The value was in agreement with the electronic conductivity of the Cu_2_Se sample conventionally sintered at 973 K for 20 h [36]. Analogously, the resistivity of Cu_2_Se decreased 4.1 times and the sheet resistance 3.7 times. The reason was the contacts between nanoparticles, which formed better conductive networks [37].

**Fig. 3:**
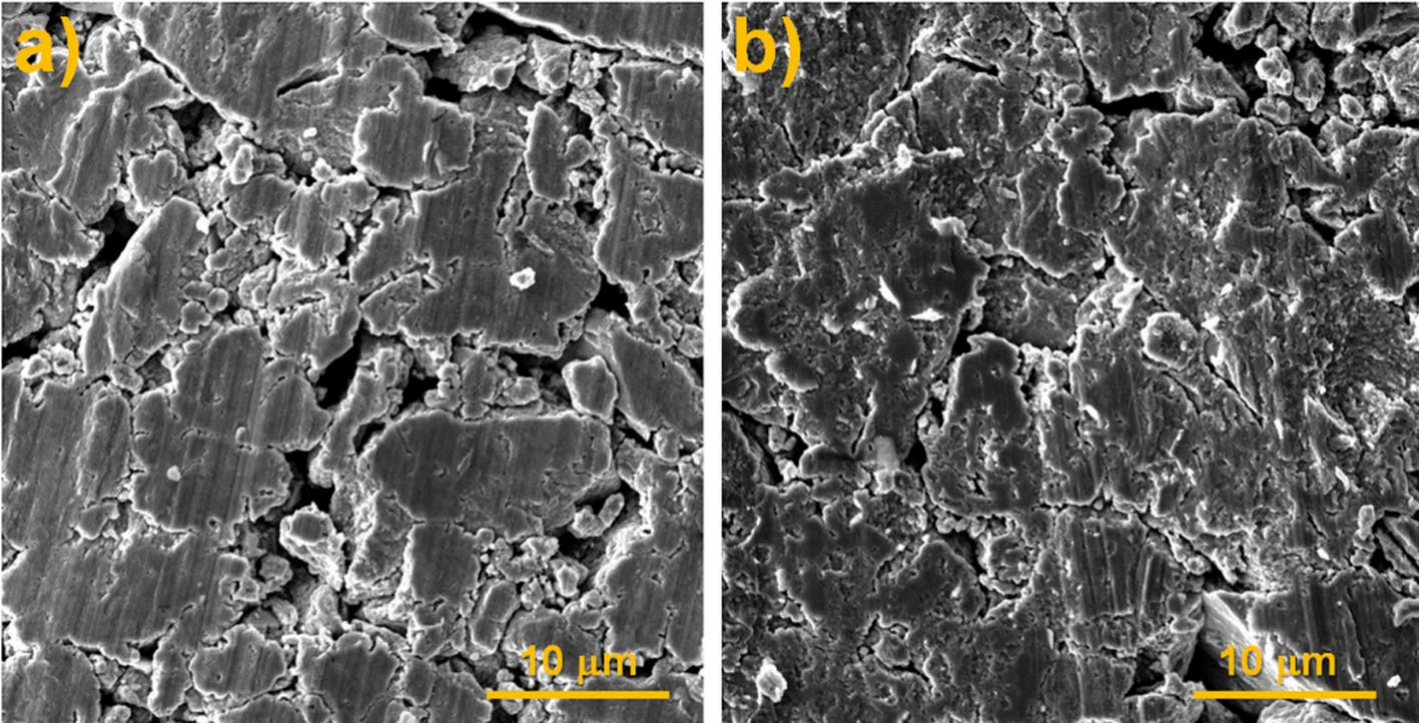
SEM images of Cu_2_Se products after applying various pressure of the tablet press: **a** 1 t, **b** 2 t

The SEM observations of the Cu_2_Se products obtained by applying the pressures of 1 and 2 t in Fig. 3 also clearly documented the decrease in porosity of Cu_2_Se/2 t due to a reduction in pore size and number. Due to the higher pressure, the grain boundaries are reduced, thereby the grain boundary scattering of carriers is also reduced and the conductivity is improved.

### The study of the thermoelectric properties of synthesized and natural Cu_2_Se

**Fig. 4:**
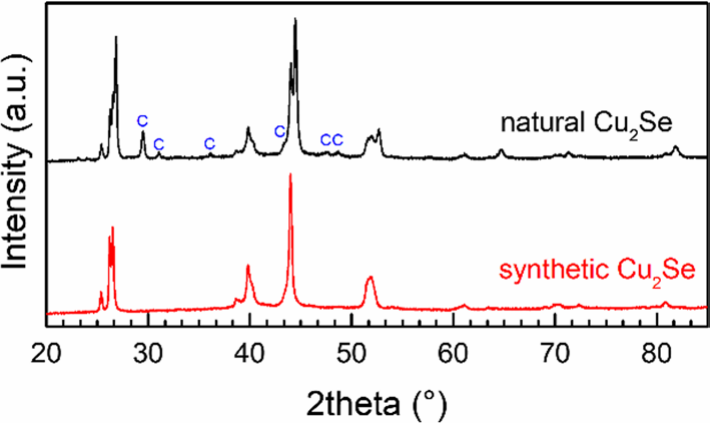
XRD patterns of synthetic and natural Cu_2_Se-berzelianite after SPS. (C–CaCO_3_, calcite: impurity phase)

The results of the Rietveld analysis showed that nanostructured synthetic Cu_2_Se prepared by high-energy ball milling crystallized in an HT orthorhombic phase [31]. After SPS, which was applied for densification of the samples for measurement of thermoelectric properties, the phase composition and crystal structure of natural (mineral berzelianite) and synthetic Cu_2_Se were unchanged which was confirmed by XRD patterns in Fig. 4.

**Fig. 5:**
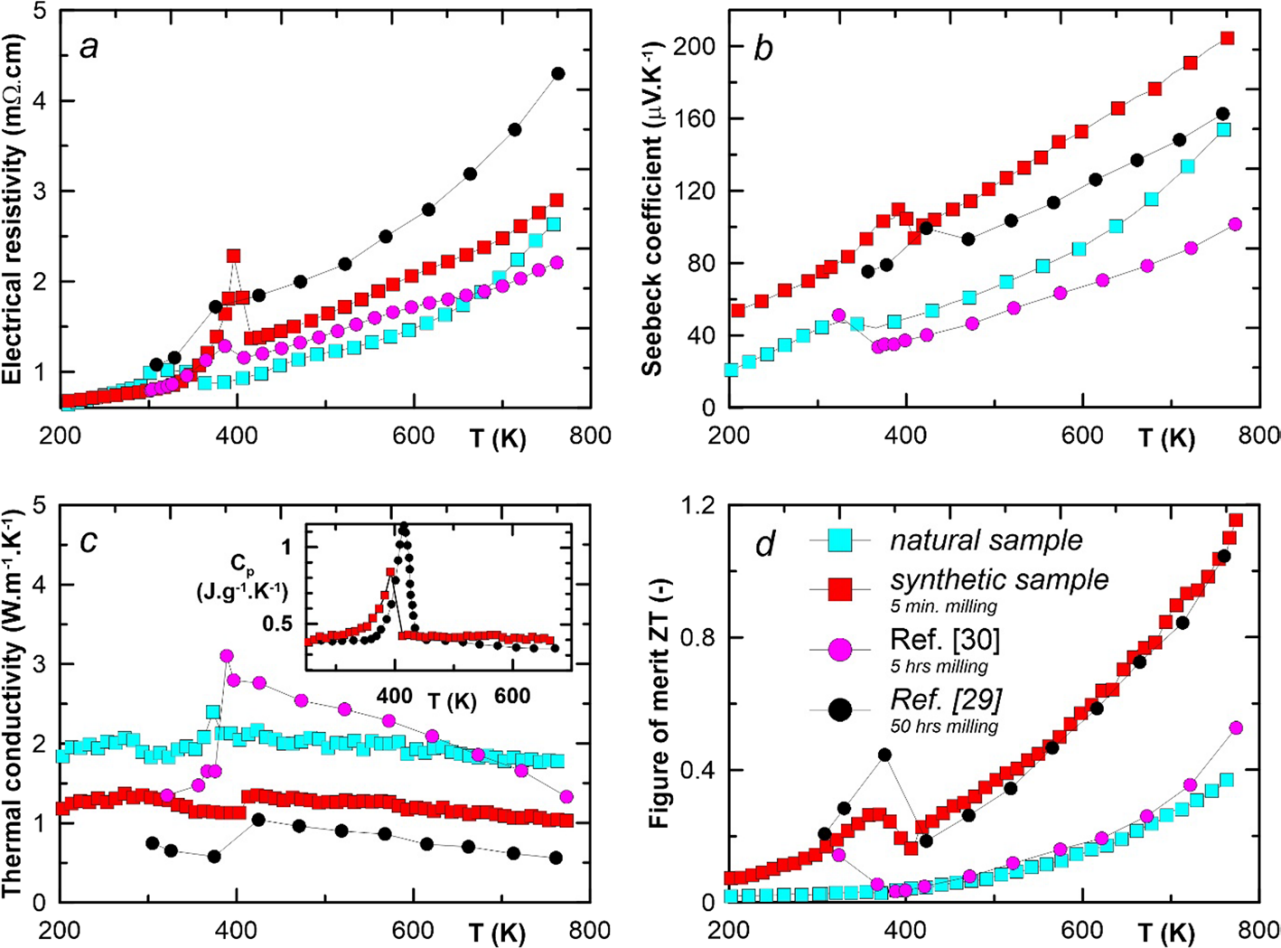
High-temperature thermoelectric properties of natural (cyan squares) and synthetic (red squares) Cu_2_Se (5 min ball milling time) as a function of temperature in the range of 200–750 K: **a** electrical resistivity *ρ*, **b** Seebeck coefficient *S*, **c** thermal conductivity *κ*, and **d** dimensionless figure-of-merit ZT. The results of the published experiments using 50 h ball milling time (black circles) [29] and 5 h ball milling time (violet circles) [30] are presented for comparison. The inset in Fig. 5c represents a comparison of the temperature dependence of specific heat (C_p_) values of our synthetic samples (red squares), which was determined by comparative method on LFA 427 (Netzsch, Germany) apparatus, with the values determined by DSC (black circles) [29]

**Fig. 6:**
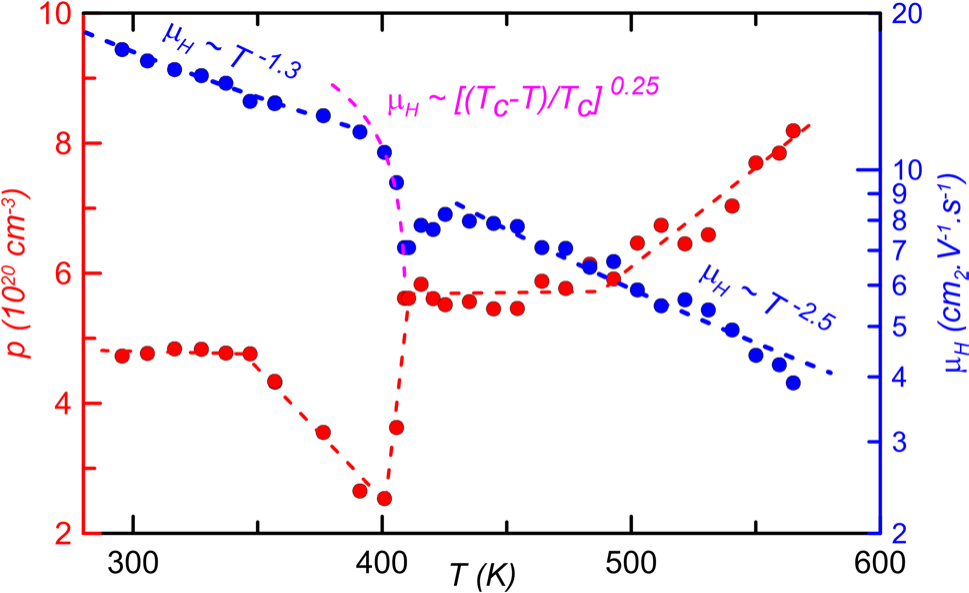
Free carrier concentration *p* (red circles) and Hall mobility *µ*_*H*_ (blue circles) as a function of temperature *T* for the synthetic Cu_2_Se sample. The red dashed line serves only as a guide to the eye. Blue dashed lines represent Hall mobility slopes for the pure α-phase (T < 360 K) and the pure β-phase (T > 410 K). The violet dashed line represents fit according to a critical power law with Tc = 410 K and the critical exponent r = 0.25

However, the superionic α–>β phase transition was reflected in the transport and thermolectric properties measurements (Fig. 5a–c) for both the synthetic and the mineral samples. For the synthetic sample was the phase transition well visible from the fluctuations of the electrical resistivity *ρ*, the Seebeck coefficient *S* and the thermal conductivity *κ* at T ~ 400 K. Much less pronounced changes in these properties can be observed for the natural sample, consisting of a mixture of polymorphs and contaminated with calcite mineral (CaCO_3_), at temperatures around 350 K. The positive values of *S* hinted that holes represented the major charge carrier for both studied samples. This fact was confirmed also by a positive sign of the Hall coefficient, *R*_*H*_ values observed for the synthetic sample. The concentration of the free carriers for this sample around 300 K started at 4.7 × 10^20^ cm^−3^ and increased up to 8.2 × 10^20^ cm^−3^ at 570 K with a steep downward fluctuation in the area below the phase transition, i.e. between 360 and 410 K (see Fig. 6). Such high free carrier concentration, the linear increase of *S* and *ρ* (except the fluctuations in the phase change region) classified both samples as degenerate semiconductors. The slopes of the temperature dependence of Hall mobility *µ*_*H*_ = *f(T)* outside of the phase change region indicated in both the pure α-phase (T < 360 K) and the pure β-phase (T > 410 K) showed a dominance of acoustic scattering mechanism. Inside the region, one can observe dramatically decreased mobility with an extremely large temperature dependence, which was according to [38, 39] fully consistent with the ideal characteristic of electron critical scattering.

The values of the dimensionless thermoelectric figure-of-merit (ZT) of the synthetic sample surpassed more than three times these for the natural sample (see Fig. 5d). It was due to the almost twice higher values of thermal conductivity of the natural samples, which can be caused by the presence of a quite high concentration of calcite impurity phase in the mineral sample (see Fig. 4) and mainly by a lower Cu stoichiometry of the sample, which is given approximately as Cu_1.8_Se. Just the difference in the stoichiometries of both samples explained also the observed changes between *ρ *and *S* values of both samples. The maximal value of the ZT parameter, i.e. ZT = 1.15 at 770 K, was well comparable with the previously reported ones for Cu_2_Se composition [27, 29]. One of the highlights of this work was the preparation of high-quality Cu_2_Se material, synthesized in a very high yield (100%) within a very short reaction time (5 min).

Table 2 compares the ZT value of mechanochemically synthesized Cu_2_Se with published ZT values and preparation conditions of Cu_2_Se synthesized by various and combined techniques. It is obvious that the highest ZT values were achieved just by the Cu_2_Se products synthesized by milling-mechanical alloying, in comparison with techniques that are also multi-step and especially more temperature-demanding. Our mechanochemical synthesis process was the fastest among all the mentioned techniques, one-step, one-pot and performed at room temperature with the ZT value of > 1. In addition, the main advantage of such synthesis is easy scaling up using the industrial vibratory mills, e.g. ESM 324-1 ks (Siebtechnik, Germany), capable of producing up to tons of material per year. Further research and testing is still needed for verification.

**Table 2:** Comparison of ZT values of Cu_2_Se synthesized by various methods and conditions

Method	Temperature/ Time	ZT	Year/Ref
Magnetron sputtering & Thermal evaporation deposition	200 °C/– 200 °C/1 h	0.35 @ 298 K	2021/[40]
Co-sputtering & Annealing	RT*/30 min 300 °C/30 min	0.42 @ 548 K	2022/[41]
Microwave-assisted hydrothermal	180 °C/3–30 min	0.56 @ 673 K	2023/[15]
Melt-annealing & Sintering	743–1473 K/10–24 h 793–1093 K/10 min	1.34 @ 873 K	2023/[25]
Microwave heating &	RT/10 min	0.65 @ 523 K	2024/[23]
Sintering	773 K/30 min		
Cold sintering & Post-annealing	473 K/1 h 823 K/1 h	0.65 @ 800 K	2024/[42]
Ball milling &	RT/10–30 h	1.35 @ 770 K	2012/[27]
Hot pressing	400–700 °C/–		
Ball milling &	RT/50 h	1.10 @ 770 K	2015/[29]
Spark plasma sintering	873 K/3 min		
Ball milling &	RT/5 h	0.60 @ 770 K	2016/[30]
Spark plasma sintering	550 °C/5 min		
Mechanical alloying & Pressing & Sintering	RT/10 h 573–973 K/1 h	1.44 @ 773 K	2023/[43]
Mechanosynthesis & Spark plasma sintering	RT/5 min 500 °C/15 min	1.15 @ 770 K	[This work]

^*^RT-room temperature

### The study of the electrochemical properties of synthesized Cu_2_Se as a cathode for sodium-ion battery

**Fig. 7:**
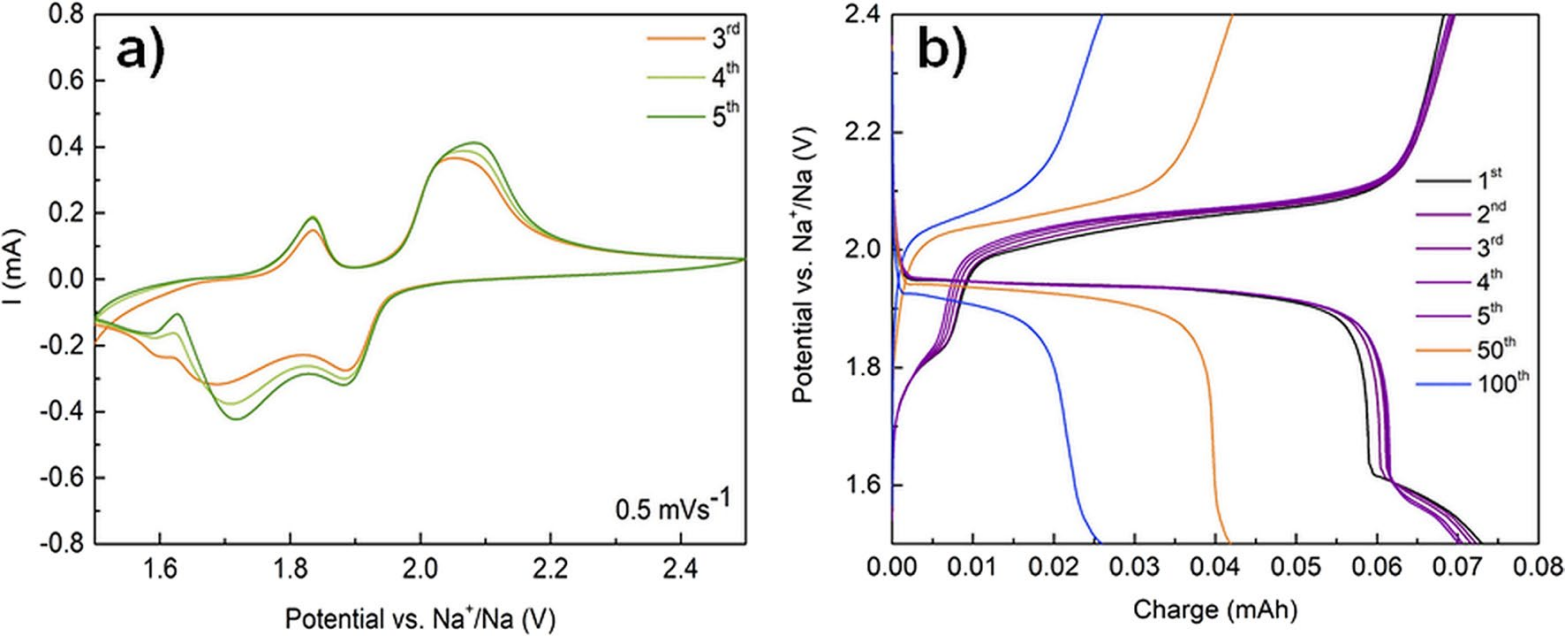
**a** CV curves of the Na/NaClO_4_/Cu_2_Se cell between 1.5 to 2.5 V vs. Na^+^/Na at the scan rate of 0.5 mVs^−1^; **b** Typical charge–discharge voltage profiles of the initial 100 cycles of the Na/NaClO_4_/Cu_2_Se battery at a constant current rate of 10 mA between 1.5 and 2.4 V

In Fig. 7a) are presented the cyclic voltammetry (CV) curves of mechanochemically synthesized Cu_2_Se electrode-cathode at the scan rate of 0.5 mV s^−1^ from 1.5 to 2.5 V. As can be seen, the electrode showed quasi-reversible sodium ion insertion/extraction ability. During these five reduction processes, two obvious cathodic peaks at about 1.6 and 1.8 V were observed. For the corresponding oxidation processes, two anodic peaks were observed at about 1.8 and 2.1 V. The cathodic peak at about 1.8 V and anodic peak at 2.1 eV were in agreement with the measurement for the Cu_2_Se electrode fabricated by a single-step postselenized method exposing the surface of the copper grid to selenide vapour in a vacuum chamber at 400 °C according to [44]. A voltage polarization of around 0.3 V between the charge and discharge profiles is suitable for practical application.

The cyclic performance of the Cu_2_Se electrode together with the reversibility of Na intercalation was tested by GCLP measurements. In Fig. 7b) the first 5, 50th, and 100th charge–discharge voltage profiles of the Na/NaClO_4_/Cu_2_Se battery cycled between 1.5 and 2.4 V at a constant current of 10 mA are shown. From these measurements, an even lower polarization value of 0.1 V was confirmed, since the plateau recorded during discharge was around 1.95 V and the charging plateau was around 2.05 V. These plateaus are related to Na-ions intercalation into Cu_2_Se and its electrochemical reaction. The first results implied that mechanochemically synthesized Cu_2_Se could be used as a promising intercalation electrode for sodium-ion batteries (SIBs).

**Fig. 8:**
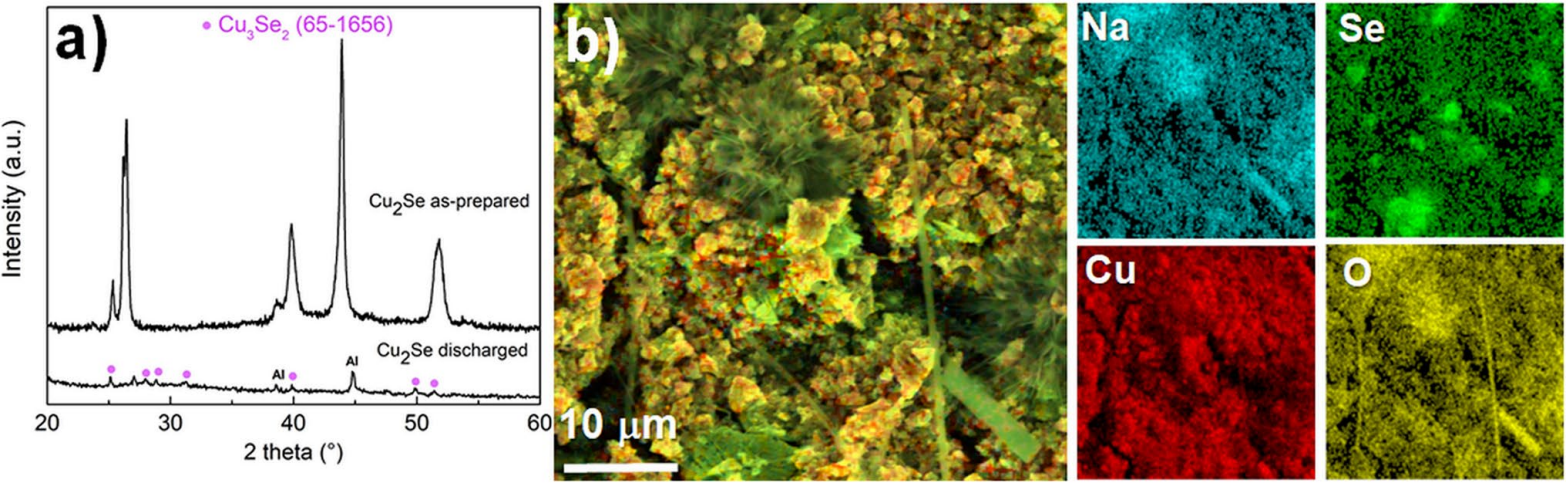
**a** The ex-situ XRD patterns of Cu_2_Se cathode material as-prepared and after discharging during the 100 cycles of cell; **b** SEM microphotograph of discharged Cu_2_Se cathode with element mapping for Na, Se, Cu and O

Ex-situ XRD analysis of discharged Cu_2_Se cathode was performed to identify the products of electrochemical reaction with sodium reported by [44]. Figure 8a) showed patterns of mechanochemically prepared Cu_2_Se cathode (as-prepared) and after discharging to 1.5 V. By comparing these patterns, it was clear that there was a total conversion of Cu_2_Se and its diffraction peaks disappeared after discharging. Two aluminium peaks come from the Al-foil substrate. However, the peaks of Na_2_Se and Cu have not appeared as products of the electrochemical reaction between Cu_2_Se and Na^+^, probably due to the formation of nanoparticles, which could not be identified using the XRD technique. In addition, unprominent Cu_2_Se_3_ (ICDD PDF 65-1656) peaks belonging to the electrochemical reaction by-product appeared, which is known as a low-temperature phase in the Cu-Se system stable at room temperature [45]. This by-product arising during the discharge process can be transformed from the metastable β-Cu_2_Se through the Cu-ion migration with high mobility in the Se-sublattice [46].

Lower magnification SEM image with element mapping of the discharged Cu_2_Se cathode in Fig. 8b) demonstrated the presence of Na a Se which probably corresponded to the Na_2_Se product of the electrochemical reaction. The presence of copper can correspond to elemental Cu but also to Cu_2_Se_3_ a by-product of the electrochemical reaction which was also indicated by the XRD pattern of the discharged cathode in Fig. 8a).

## Conclusion

In this work, the properties of mechanochemically prepared Cu_2_Se, which have the potential to be applied in practice, were investigated for the first time. The thermal analysis confirmed the complete conversion of the precursors of the mechanochemical reaction Cu and Se to the Cu_2_Se product after only 5 min of milling and its thermal stability. The reduction of its porosity due to press pressure had a positive impact on the increase in conductivity and the decrease in sheet resistance and resistivity, which is an interesting technological parameter for the fabrication of electrodes from the semiconductor synthesized in this way. Competitive values of the ZT parameter 1.15 at 770 K were achieved by the rapid mechanochemical reaction for the synthetic Cu_2_Se sample. The first results of electrochemical measurements of the Na/NaClO_4_/Cu_2_Se cell indicated that the mechanochemically synthesized Cu_2_Se has a promising potential for use as an intercalation electrode in SIBs.

## Data Availability

The authors declare that the data supporting the findings of this study are available within the paper. Should any raw data files be needed in another format they are available from the corresponding author upon reasonable request. Source data are provided in this paper.
